# Spinal intradural extramedullary tumors: microscopic keyhole resection with the focus on intraoperative neurophysiological monitoring and long-term outcome

**DOI:** 10.1186/s13018-023-04074-z

**Published:** 2023-08-14

**Authors:** Bofei Yu, Yuhua Xiao, Hanhao Dai, Yunlong Yu, Yuan Lin, Jie Xu

**Affiliations:** 1 Division of Spine Surgery and Electrophysiological Center, Department of Orthopedics, Fujian Provincial Hospital, Fujian Medical University, No. 134, East Street, Fuzhou, 350001 Fujian China; 2 Shengli Clinical Medical College of Fujian Medical University, Fuzhou, Fujian China

**Keywords:** Intradural tumor, Keyhole surgery, Long-term outcome, Intraoperative neurophysiologic monitoring

## Abstract

**Objective:**

Spinal schwannomas (SS) and spinal meningiomas (SM) account for most intradural extramedullary (IDEM) tumors. These tumors are usually benign lesions, which generally respond favorably to surgical excision. Few studies up to now tried to determine the long-term outcome after minimally invasive surgery (MIS) with multimodal intraoperative neurophysiological monitoring (IONM) for IDEM tumors. The aim of this study was to present one of the largest case series with special regard to IONM findings and long-term outcome after MIS-keyhole surgery with a tubular retractor system.

**Methods:**

Between January 2013 and August 2018, 87 patients with IDEM tumors who underwent tumor removal surgery via MIS-keyhole approach under multimodal IONM were retrospectively reviewed. The neurological status was assessed using a modified McCormick grading scale pre- and postoperatively. Multimodal IONM consisted of motor evoked potentials (MEP), somatosensory evoked potentials (SEP), and electromyography (EMG). Both short-term and long-term clinical evaluations as well as patients’ medical files were retrospectively analyzed.

**Results:**

Surgeries were performed for resection of SS in 49 patients and SM in 38 patients. Tumor locations were cervical in 16.1%, thoracic in 48.3%, thoracolumbar in 4.6%, lumbar 31%. Critical IONM changes were detected in 9 operations (10.3%) in which there were 2 SEPs, 5 MEPs, and 2 EMG events. Three IONM changes (2 MEPs, 1 EMG) were turned out to be transient change in nature since they were resolved in a short time when immediate corrective actions were initiated. Six patients with permanent IONM changes (2SEPs, 3MEPs, 1EMG event), all deficits had resolved during hospitalization or on short -term follow-up evaluation. Sensitivity, specificity, and positive and negative predicted values of IONM were 100, 96, 67, and 100%, respectively. Gross total resection rate was 100%, and a stable or improved McCormick grade exhibited in all patients. No tumor recurrence and no spinal instability were found in the long-term follow-up evaluation (mean 5.2 ± 2.9 years postoperatively). Overall, 94% of patients were either satisfied or very satisfied with their operation, and 93% patients reported excellent or good general clinical outcome according to Odom’s criteria.

**Conclusion:**

MIS-keyhole surgery with multimodal IONM for IDEM tumors enables a high level of satisfaction and a satisfying long-term clinical and surgical outcome.

## Introduction

Spinal schwannomas (SS) and spinal meningiomas (SM) represent the most common intradural extramedullary (IDEM) lesions. Surgical resection is the preferred treatment option with the goal of gross tumor removal while minimize neural injury [1, 2]. For this reason, application of intraoperative neurophysiological monitoring (IONM) during IDEM tumor removal surgery to reduce the incidence of neurological complications and iatrogenic damage has been reported in the literature [3–7], but the role of multimodal IONM in minimally invasive surgery (MIS) for IDEM tumors has not been adequately addressed. We have previously reported the surgical resection of spinal dumbbell tumors and thoracic spinal meningiomas using MIS-keyhole technique through a tubular retractor system [8, 9]. In the current paper, we report our experience by presenting one of the largest case series with special regard to IONM findings and long-term outcome after such MIS technique.

## Methods

### Patients

We retrospectively reviewed 87 consecutive patients who were surgically treated at the Division of Spine Surgery and Electrophysiological Center, Department of Orthopedics, Fujian Provincial Hospital, between January 2013 and August 2018 for IDEM tumors. Inclusion criteria were primary intradural extramedullary tumors, and cases undergoing microsurgical keyhole resection with multimodal intraoperative neurophysiological monitoring. Exclusion criteria were those with a history of spinal trauma or surgery at the same segment, and those patients with incomplete follow-up data. The patients’ medical records and radiological studies were extracted from the electronic medical record system. The study protocol was approved by the Ethics Committee of the Fujian Provincial Hospital, and informed consent was obtained from all enrolled participants.

### Surgical procedures and IONM

All patients were placed in the prone position, and surgical procedures were performed by the same surgical team using the microscopic keyhole approach through a tubular retractor (Medtronic Sofamor Danek, Memphis, Tennessee, USA, or Johnson & Johnson, New Brunswick, New Jersey, USA).

Anteroposterior and lateral intraoperative fluoroscopy were used to identify the target level. An approximately 20–25-mm paramedian linear skin incision was made according to the lateral projection of the lesion. The subcutaneous tissues and dorsal fascia were stripped initially, and then, a set of dilatators (DePuy Spine, Inc.) were inserted to separate the paraspinal muscles bluntly. The dilators were removed, a tubular retractor with a diameter of 25 mm was fixed as a channel, and the correct position of the tubular retractor was confirmed using intraoperative fluoroscopy again.

Once the tubular surgical path was established, the operating microscope (Leica M525 F40; Leica Microsystems GmbH, Wetzlar, Germany) was introduced. Surgical procedures for IDEM tumors (including SS and SM) were described in detail in our previous published papers [8, 9]. Total intravenous anesthesia was induced for all patients. There were no muscle relaxants administered during whole procedure except initial anesthesia induction. All anesthesiologists involved in major orthopedic surgery participated in intraoperative anesthesia skill training related to neuroelectrophysiological monitoring.

Multimodal IONM including MEP, SEP, EMG during whole surgical procedure was performed by a technician trained in IONM with NIM-Eclipse system (Medtronic, USA).

#### Somatosensory evoked potential (SEP)

The stimulation position of SEP was determined by different operations; specifically, SEP was monitored at the median nerve of the upper limb and the posterior tibial nerve of the lower limb. Upper and lower limb SEP needs to be monitored above the conus medullaris, while SEP is not monitored below the conus medullaris.

Constant current stimulation is useful in monitoring SEP, and the stimulations also performed with constant voltage stimulation. C3 'and C4' are selected for upper limb, CZ 'is selected for lower limb, and FZ is selected as reference electrode.

#### Motor evoked potential (MEP)

C3 and C4 are the stimulation points of MEP. The recording electrodes are bilateral thenar muscles for upper extremity representation, and anterior tibialis or abductor hallucis brevis muscles for lower extremity representation as well as the muscles corresponding to the surgical segment.

#### Electromyography (spontaneous free-running EMG, sEMG or triggered EMG, tEMG)

Generally, L1 and L2 were recorded in the adductor major, L3 and L4 in the quadriceps femoris, L4 and L5 in the tibialis anterior, and S1 in the gastrocnemius. The EMG in a resting state appears as a straight line, while an EMG burst is accompanied by a sudden appearance of intense waves.

### Alarm criteria for evoked potentials

The specific IONM alarm criteria in our institution are a more than 50% reduction in MEP amplitude, and/ or a significant reduction of SEP amplitude ≥ 50% or a more than 10% of N20 or P37 latency prolongation for SEPs, and /or significant spontaneous EMG activity especially during or after surgical manipulation. The surgical team was promptly informed, and the surgical procedures were stopped temporarily if any significant IONM changes occurred.

### Definitions of IONM results

#### True positive

Patients suffered from new neurological deterioration that was positively correlated to relevant IONM findings, or a case where a significant signal deterioration improved to the baseline value after a specific intraoperative corrective actions were initiated.

#### True negative

Normal IONM findings accompanied by the absence of a new postoperative neurological deficit.

#### False positive

Patients emerged from surgery with neurologically intact, although significant attenuation or abolishment in IONM data occurred.

#### False negative

Patients emerged from surgery with a new postoperative neurological deficit, but IONM findings turned out to be normal.

### Data collection and outcome evaluation

In our department, postoperative clinical visits were routinely scheduled at 1, 3, 6, and 12 months, and every 6 months thereafter. The neurological state of each patient was evaluated according to the classification of McCormick (Table 1) [10] before and after surgery as well as at the time point of the last clinic visit. New or worsened sensorimotor deficits, possible recurrence and complications were recorded postoperatively. Gross total resection was defined as complete tumor resection based on both intraoperative microscopic findings and postoperative/follow-up spinal MRI images. The patients’ postoperative general clinical situation was evaluated using Odom’s criteria [11]. The degree of patients’ satisfaction regarding the general surgical outcome was assessed using the criteria described by Hamilton et al. [12].

**Table 1:** Modified McCormick grading scale

Grade	Modified McCormick grading scale
I	Intact neurologically, normal ambulation, minimal dysesthesia
II	Mild motor or sensory deficit, functional independence
III	Moderate motor/sensory deficit; limitation of function; independent with external aid
IV	Severe motor or sensory deficit, May or may not function independently
V	Severe deficit. Requires wheelchair or cane/brace with bilateral upper extremity impairment. Usually not independent

## Results

A total number of 87 patients (33 M, 54 F) was included for the final analysis. All patients underwent successful 1-stage surgical resection of intraspinal tumors without conversion to open traditional surgery. There were no deaths or 30-day readmission in our series. Surgeries were performed for resection of SS in 49 patients (21 with dumbbell shape) and SM in 38 patients (11 ventral or ventrolateral, 27 dorsal). As about localization, tumor locations were cervical in 14 patients (16.1%), thoracic in 42 patients (48.3%), thoracolumbar in 4 patients (4.6%), lumbar in 27 patients (31%). The complication rate was 11.5%, including 3 cerebrospinal fluid leakages, 1 superficial wound infection, and 6 cases of neurological deterioration. Gross total resection (GTR) rate was 100%, and no tumor recurrence or spinal instability was found in the long-term follow-up evaluation (mean 5.2 ± 2.9 years postoperatively). No patient has been lost to follow-up. Patients’ baseline clinical and demographic data, as well as surgery-related complications are shown in Tables 2 and 3. Sensitivity, specificity, and positive and negative predicted values of IONM were 100, 98, 67, and 100%, respectively (Table 4). Transient IONM changes were detected in 3 operations (3.4%) in which there were 2 MEPs and 1 EMG event. Waveform attenuation on MEPs was detected in two patients that were related to hypotension. Significant spontaneous EMG activity was detected in 1 patient that was caused as a result of spinal cord manipulation during dissection of the tumor masses. All alerts were resolved when intraoperative corrective actions were initiated, such as transient stopping the procedure, raising blood pressure, increasing body temperature, and irrigation of the surgical field with warm saline solution or administration of steroidal treatments.

**Table 2:** Baseline clinical and demographic data of 87 patients with IDEM tumors

Variable	Total
Mean age in yrs ± SD	56.8 ± 11.3
*Sex*	
Female	54 (62%)
Male	33 (38%)
*Tumor location*	
Cervical	14 (16.1%)
Thoracic	42 (48.3%)
Thoracolumbar	4 (4.6%)
Lumbar	27 (31%)
*Average tumor volume*	
Spinal schwannomas	2.3 × 1.8 × 1.1 cm
Spinal meningiomas	1.9 × 1.4 × 0.7 cm
*Symptoms*	
Local pain	11 (12.6%)
Radicular pain	28 (32.2%)
Paresthesia	54 (62.1%)
Motor deficit	67 (77%)
Gait impairment	15 (17.2%)
Urinary incontinence	2 (2.3%)
*Modified McCormick scale at admission*	
I	25
II	39
III	14
IV	9
V	0
*Pathology*	
Spinal schwannomas	49
Spinal meningiomas	38
Mean follow-up in yrs ± SD	5.2 ± 2.9

**Table 3:** Degree of surgical removal and surgery-related complications

Degree of surgical removal	Total
GTR	87 (100%)
*Complications*	10 (11.5%)
30-d mortality	0
Neurological deterioration	6 (6.9%)
Wound infection	1 (1.2%)
Postoperative hematoma	0
CSF leak	3 (3.4%)
DVT/PE event	0

GTR, gross total resection; CSF, cerebrospinal fluid; DVT, deep venous thrombosis; PE, pulmonary embolism

**Table 4:** IONM sensitivity and specificity of the group

Neurologic status	Stable IONM	IONM deterioration	Sum
Stable	78	3	81
Deterioration	0	6	6
Sum	78	9	87
Sensitivity	100%		
Specificity	96%		
PPV	67%		
NPV	100%		

IONM, intraoperative neurophysiological monitoring; PPV, positive predictive value; NPV, negative predictive value

Permanent IONM changes were noted in 6 patients (2SEPs, 3MEPs, 1EMG event), all 6 patients showed immediate postoperative deterioration, two resolved completely at discharge, four impairments remained at discharge, two of four patients resolved by the 3-month follow-up evaluation.

A 68-year-old man with thoracic spinal meningiomas presented with difficulty ambulating, preoperative severe lower limb weakness (right: MRC Grade 2, left: Grade 3) had improved in power (right: MRC Grade 4, left: Grade 4) at the 3-month follow-up evaluation, however, he developed urinary retention at that clinical visit, finally, after attended a urology clinic visit in our institute, he was found to have severe benign prostatic hypertrophy, suggesting a contribution from BPH itself rather than surgery.

Another obese patient's preoperative symptoms were numbness of both lower limbs for 1 year and back pain for 3 months. The back pain disappeared immediately after operation, but the numbness symptoms of both lower limbs were not significantly relieved. After eliminating the possibility of nerve compression in the spinal canal, it is suggested that active measures should be taken to control blood sugar and lose weight, the patients accepted the suggestion gladly. At 1-year follow-up, the numbness of both lower limbs was partially relieved, but the patient was satisfied with the curative effect of the operation and accepted the current situation.

A stable or improved McCormick grade exhibited in all patients; specifically, at the 3-month follow-up, 72 patients (82.8%) exhibited improved neurological status after surgery, 15 patients (17.2%) remained unchanged. At the 12-month follow-up, 79 patients (90.8%) showed improved neurological status, and 8 patients (9.2%) remained stable (normal). At the latest follow-up visit, we found that the rate of improvement was consistent with those before, no worsening of the neurological status was observed.

During the short-term follow-up period (within 1 month after surgery), according to Odom’s criteria, 64 patients (73.5%) having an excellent or good outcome, 23 (26.5%) having a fair outcome, and no patients were rated as “poor outcome.” At the latest follow-up examinations, all patients reported a stable or improved general clinical outcome, with 81 patients (93%) having an excellent or good outcome, 6 (7%) having a fair outcome, and no patients were recognized as “poor outcome” (Table 5).

**Table 5:** General clinical outcome evaluation at both short-term and long-term follow-up period, according to Odom’s criteria

Outcome	Total	
	Short-term outcome	Long-term outcome
	(Within 1 month postop, *n* = 87)	(3–8 yrs postop, *n* = 87)
Excellent	21 (24.1%)	28 (32.2%)
Good	43 (49.4%)	53 (60.9%)
Fair	23 (26.5%)	6 (6.9%)
Poor	0	0

Odom’s criteria, Excellent: all preoperative symptoms relieved, abnormal findings improved; Good, minimal persistence of preoperative symptoms, abnormal findings unchanged or improved; Fair, definite relief of some preoperative symptoms, other symptoms unchanged or slightly improved; and Poor, symptoms and signs unchanged or worse

At the latest outpatient follow-up visit, 94% of patients were reported to be either satisfied or very satisfied with their surgical outcome: 92% said they would undergo the surgery again if they had a choice and 88.5% would recommend the operation to their friends or family members (Table 6).

**Table 6:** Patient satisfaction survey (n, %)

Question measuring satisfaction with Likert Scale	Very satisfied	Satisfied	Dissatisfied	Very dissatisfied	Unsure
*Satisfaction items*					
Are you satisfied with your operation? (four point scale)	17 (19.5%)	65 (74.7%)	2 (2.3%)	1 (1.2%)	2 (2.3%)

Question regarding specific aspects of surgery	Excellently	Very well	Well	Fairly	Poorly	Don’t know
How well did the surgery relieve your pain?	21 (24.1%)	60 (69%)	3 (3.4%)	2 (2.3%)	1 (1.2%)	0
How well did the surgery increase your ability to perform regular activities?	15 (17.2%)	53 (61%)	11 (12.6%)	8 (9.2%)	0	0
How well did the surgery allow you to perform heavy work or sport activities? (e.g. carrying heavy grocery bags during marketing, or in day to day activities, etc.)	19 (21.8%)	48 (55.2%)	16 (18.4%)	3 (3.4%)	0	1 (1.2%)
How well did the surgery meet your expectations?	67 (77.1%)	15 (17.2%)	3 (3.4%)	2 (2.3%)	0	0
Can you rate your overall hospital experience?	50 (57.5%)	22 (25.3%)	10 (11.5%)	4 (4.5%)		1 (1.2%)

Question regarding overall satisfaction	Definitely yes	Possibly yes	Probably not	Certainly not	Not sure
After knowing what this operation is about, if you were given a choice again, would you have this operation again?	80 (92%)	2 (2.3%)	0	1 (1.2%)	4 (4.5%)
Would you recommend the surgery to a friend/family member?	77 (88.5%)	5 (5.7%)	2 (2.3%)	1 (1.2%)	2 (2.3%)

**Fig. 1:**
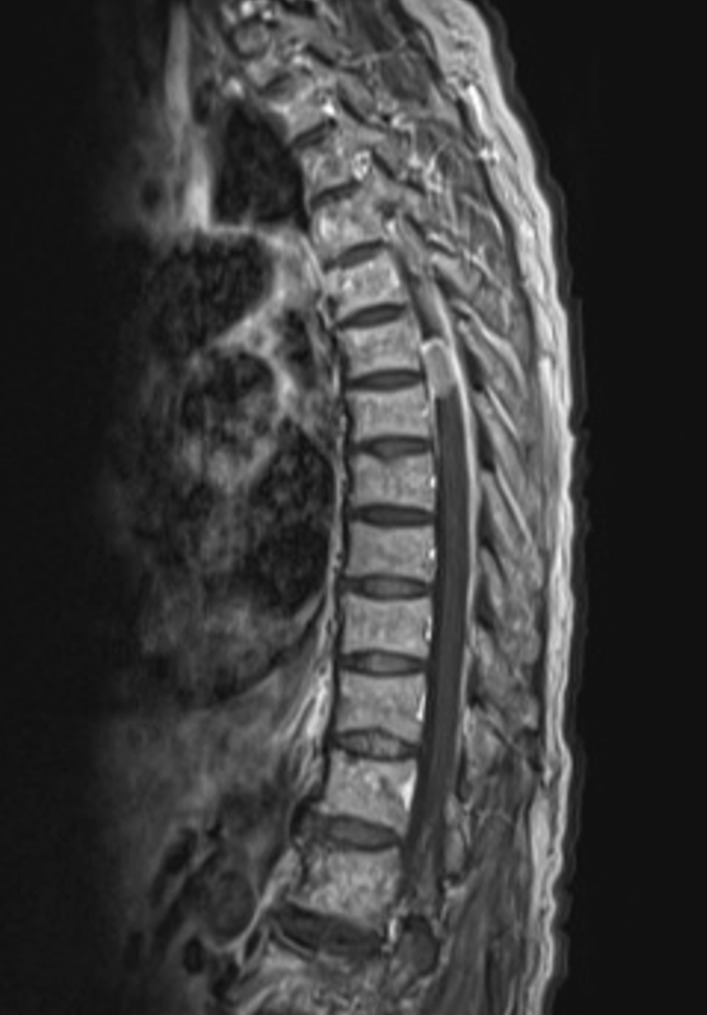
A 61-year-old woman presented with a 4-month history of numbness and pain in both lower limbs that were nonresponsive to conservative treatments. Preoperative sagittal magnetic resonance image demonstrated a large oval-shaped mass at T6-7 level

**Fig. 2:**
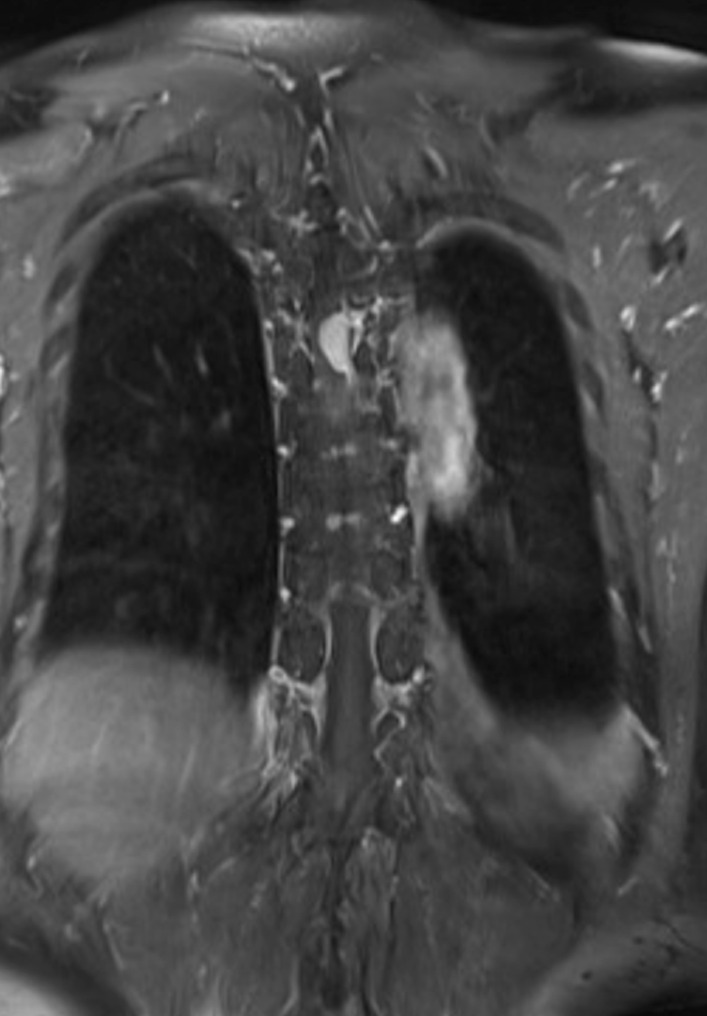
Coronal magnetic resonance image demonstrated a large oval-shaped mass at T6-7 level

**Fig. 3:**
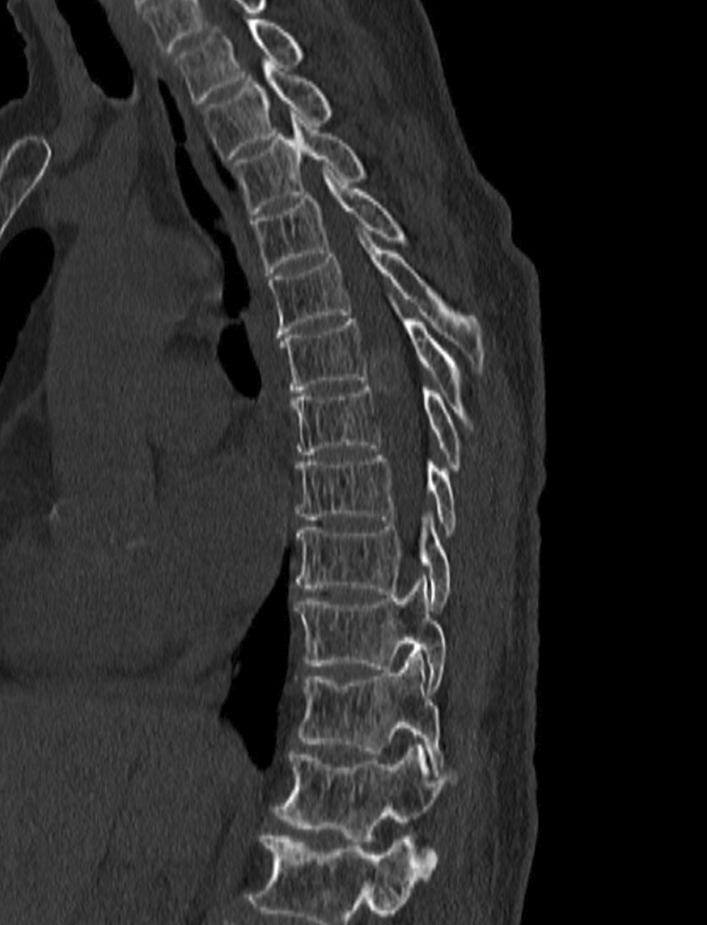
Computed tomography demonstrated a large oval-shaped mass with mild calcification at T6-7 level

**Fig. 4:**
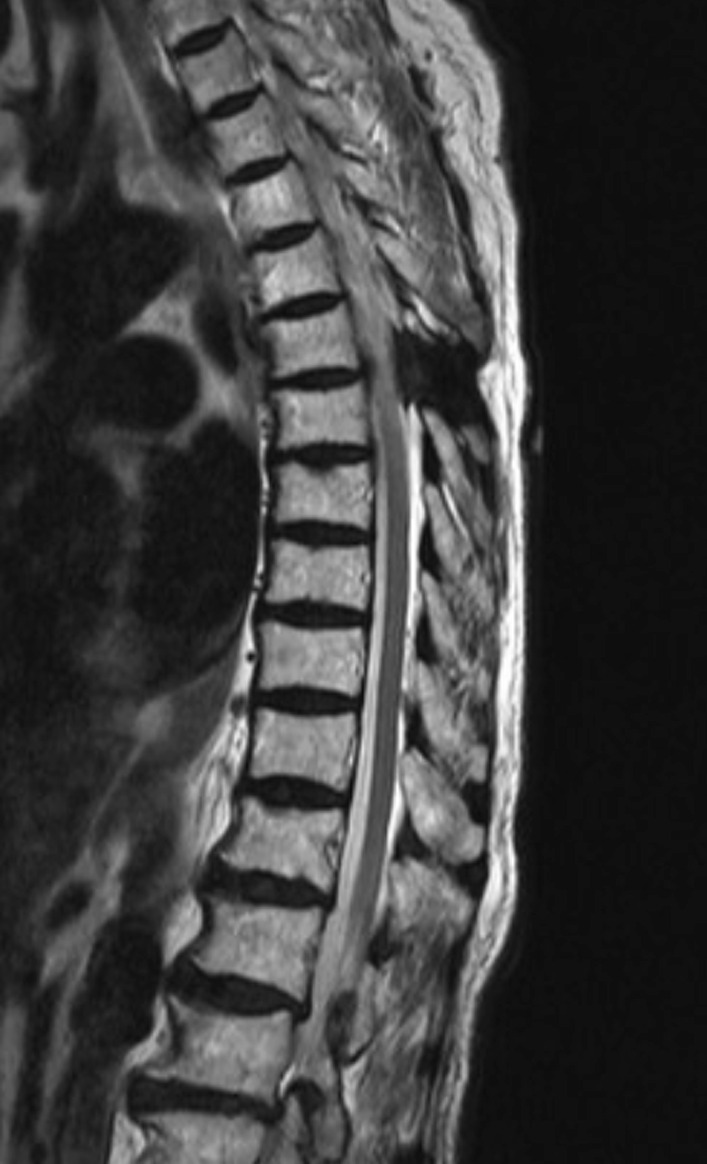
The patient underwent tumor removal surgery using the microscopic keyhole technique under multimodal IONM, and complete resection was achieved

**Fig. 5:**
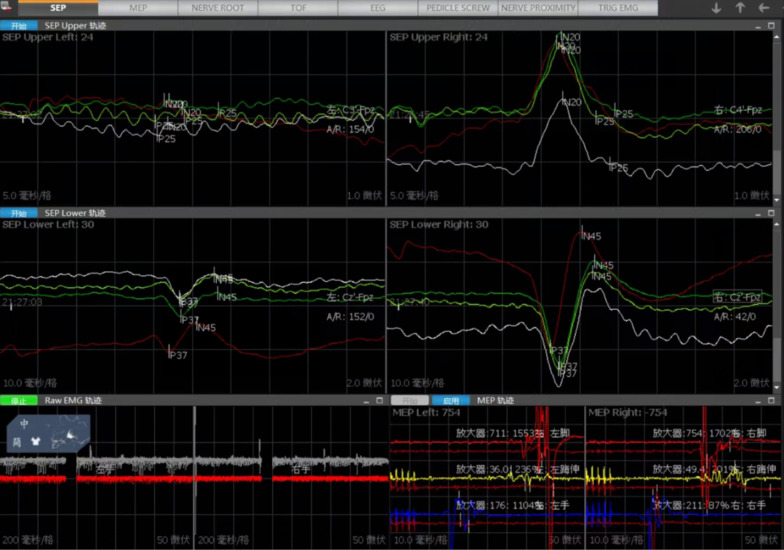
Evoked potentials remained stable throughout the surgical procedure

A typical case was shown in Figs. 1, 2, 3, 4 and 5.

## Discussion

Although spinal surgery has advanced tremendously in recent decades, IDEM tumor removal surgery still carries high risk in postoperative iatrogenic neurological injury. To address this issue, application of intraoperative neurophysiological monitoring during IDEM tumor removal procedure has gained increasing popularity across the world. Traditional open laminectomy is the most commonly approach performed by spine surgeons for resection of these tumors; recently, application of MIS techniques in the treatment of IDEM tumors and other intradural pathologies have been reported by several authors [13–20]. However, literature data about the role of multimodal IONM in MIS for IDEM tumors are few. Therefore, we retrospectively reviewed 87 consecutive spinal tumors operated by the MIS-keyhole technique using the tubular retractor system with multimodal IONM. To the best of our knowledge, this is one of the largest case series to evaluate the clinical efficacy on patients undergoing IDEM tumor removal surgery via MIS, with emphasis on the role of multimodal IONM findings and long-term outcome.

With the application of IONM in a wide range of spinal surgical procedures, single-modality IONM technique has proven to be insufficient due to the complexity of both ascending and descending nerve conduction pathways [21]. In our institute, spinal surgeries involving potential compromise of spinal cord and nerve root function were monitored routinely by multimodality IONM including SEP, MEP, and EMG. The current study adds to the literature showing that multimodality IONM techniques can prove feasible and useful for intradural pathology [4, 22–24]. In our series, multimodal IONM during MIS-keyhole tumor excision surgery demonstrated a high level of accuracy, with sensitivity and specificity as high as 100% and 96%, respectively, PPV of 67%, and NPV of 100%. All parameters except PPV show better or similar results compared with previous studies [4–6]. The difference of PPV data might be attributed to different morbidity rate. Recently, Ghadirpour et al. [5] reported on 108 patients who underwent IDEM tumor removal surgery using multimodal IONM (SEP + MEP + D-wave), and the IONM data reported in their series were similar to our results, with a specificity of 97%, PPV of 67%, and NPV of 100%. However, note that the sensitivity they reported was lower than ours with 85.7%. Our paper could not provide any relevant data about the accuracy of the D-wave in IDEM tumor removal surgery, since D-wave monitoring was not performed in our institute due to equipment and technical reasons.

High sensitivity and specificity are important guarantee to protect the integrity of nerve function and ensure the curative effect of operation.

There were no false-negative alerts in our group, while false-positive warnings were noted in 3 operations (3.4%) in which there were 2 MEPs that were related to hypotension. Such two alerts were resolved when hypotension was corrected by anesthesiologist, which is in line with previous studies [25, 26]. Therefore, we insist that all electrophysiological data must always be interpreted on the premise of excluding all possible technical and anesthesiological reasons, at the same time, constant and close communication between surgeons and anesthesiologists play a vital role during whole IONM procedure.

Regarding the influence of IONM on postoperative neurological status and extent of resection, Formo et al. [16] presented their initial experience on 83 patients with IDEM spinal tumors who underwent MIS through a MAST-QUADRANT system. In their series, 4 (4.9%) patients experienced neurological deterioration and gross total resection was achieved in 87% of cases. Furthermore, Wong et al. [15] operated on 27 IDEM tumors using minimally invasive expandable retractor, they reported excellent outcomes with a 92.6% rate of GTR and no perioperative neurological deterioration. Recently, Hernandez et al. [19] retrospectively reviewed a cohort of eight patients who underwent unilateral hemilaminectomy using a Williams retractor, GTR was achieved in all cases and neurologic function appeared to have improved in most patients with an average Nurick score of 2 at initial consultation decreased to 0.71 after 2.4 months follow-up. Similarly, in a study by Balasubramanian et al. [20], Forty-one consecutive spinal tumor cases were operated by the MISS-Key Hole technique using the tubular retractor system, GTR was achieved successfully in 39 cases (95.12%), and two patients with worsening of the neurological status were noted, which improved over a period of 6 months. In the present study, GTR was achieved in all cases, six patients (6.9%) experienced neurological deterioration immediately after surgery, which is well within the range of that reported for MIS surgery [15–20], and no patient was left with a permanent deficit compared with their preoperative status as a stable or improved McCormick grade exhibited in all cases in the long-term follow-up evaluation (mean 5.2 ± 2.9 years postoperatively). These results further demonstrate that intradural pathologies can be resected safely and radically via MIS approach as with open surgery.

Despite satisfactory outcomes with both a 100% rate of GTR and expected oncological goal we have achieved, what attitude towards tumor resection, being more aggressive or conversative when IONM signals deterioration were improved, which should be considered carefully. In some contexts, utilization of multimodal IONM especially triggered EMG technique in IDEM tumor resection surgery is of the utmost importance in guiding intraoperative decision-making. Given our experience, when the tumor is large and closely adheres to the nerve, the traction or electrocautery of the tumor during the operation often leads to the stimulation of the nerve root. At this time, it is difficult to distinguish the nerve and non-nerve tissue even under the microscope. In this scenario, triggered EMG may be a valuable tool since the probe stimulation can be used to guide the surgeon in setting surgical boundaries. It should be noted that long-term and frequent stimulation of nerve roots should be avoided, because the higher the amplitude of muscle wave burst, the longer the duration, suggesting that the greater the possibility of neurological deterioration after operation. In our practice, during the operation of 87 patients with IDEM tumors, the integration of spontaneous free-running EMG and triggered EMG were employed to determine the “safety zone” and avoid “risky maneuvers,” so as to achieve the preservation of neural integrity. In addition, what needs to be emphasized rather than overlooked here is surgeon experience, the senior author (Jie Xu) had more than 30 years of robust experience with microsurgery, and applied MIS technique for intradural pathology since 2007, this may be another reason for the higher rate of GTR in our series.

Another noteworthy finding in our study is that 94% patients reported good rates of satisfaction at the latest clinical follow-up visit. So far as we know, most clinical studies on spinal cord tumors have used only the neurological status scoring system for follow-up evaluation, the survey of patients' satisfaction with surgery seems to be lacking. In 2016, Chotai et al. [27] reviewed 38 IDEM tumor patients and reported that 87% (*n* = 33) achieved satisfaction with outcome 1 year after surgery; however, only 66% of these patients (*n* = 25) achieved the highest level of satisfaction (surgery met their expectations). In our previous published literature [8, 9], it has been demonstrated in detail that minimally invasive keyhole surgery has the advantages of less bleeding, shorter hospital stay, fewer drainage tubes, less internal fixation (reduced hospitalization cost) and the clinical efficacy equivalent to traditional open surgery. All these factors are associated with higher patient satisfaction in our group. Beyond that, enhanced recovery after surgery (ERAS) protocols have been gradually applied to patients undergoing major spinal surgery. Recently, Liu et al. [28] published a randomized clinical trial study illustrating their ERAS protocols on intraspinal tumor surgery, they found that a higher patient satisfaction was observed in the ERAS group compared with the controls. In this regard, we strongly agree with their opinion that MIS instead of open spine surgery when applicable is an important component of ERAS protocols. In addition, we believe that the application of IONM is also a crucial embodiment of ERAS concept in IDEM surgery.

Our study has several limitations. First, it could not provide a high level of evidence due to the retrospective nature of this research. Second, lack of patients who underwent IDEM tumor removal without IONM for comparison, prospective studies on this issue are warranted and we would be curious to see such research. Third, we only included patients with schwannomas and meningiomas, although they accounted for the vast majority of intraspinal tumors, we have minimal experience with MIS for other intraspinal lesions.

Our study, despite the limitations that we have delineated, is the first large-scale study evaluating the role of multimodal IONM findings and long-term outcome on patients undergoing IDEM tumor removal surgery via MIS. Another strength of this study is that we also examined patient reported outcome measures regarding satisfaction rates about MIS for IDEM surgery; to the best of our knowledge, this information is scarce in existing literature.

## Conclusions

With our data, this study indicates that MIS-keyhole surgery with multimodal IONM for IDEM tumors is well feasible and enables a satisfying long-term clinical and surgical outcome as well as high level of patient satisfaction, although future research with larger number of patients and longer follow-up periods is warranted.

## Data Availability

All data generated or analyzed during this study are included in this published article.
